# Atmospheric Stilling Promotes Summer Algal Growth in Eutrophic Shallow Lakes

**DOI:** 10.3390/biology10121222

**Published:** 2021-11-23

**Authors:** Wei Zou, Guangwei Zhu, Hai Xu, Mengyuan Zhu, Chaoxuan Guo, Boqiang Qin, Yunlin Zhang

**Affiliations:** Taihu Laboratory for Lake Ecosystem Research, State Key Laboratory of Lake Science and Environment, Nanjing Institute of Geography and Limnology, Chinese Academy of Sciences, Nanjing 210008, China; wzou@niglas.ac.cn (W.Z.); hxu@niglas.ac.cn (H.X.); myzhu@niglas.ac.cn (M.Z.); cxguo@niglas.ac.cn (C.G.); qinbq@niglas.ac.cn (B.Q.); ylzhang@niglas.ac.cn (Y.Z.)

**Keywords:** climate change, eutrophication, cyanobacterial bloom, light limitation, atmospheric stilling

## Abstract

**Simple Summary:**

The variability of chlorophyll *a* yields per unit nitrogen (Chl*a*/TN), or phosphorus (Chl*a*/TP) and its influencing factors were evaluated in eutrophic shallow Lake Taihu, China. The results indicated warming and longer sunshine hours promoted Chl*a*/TN and Chl*a*/TP in winter months from 2005 to 2017, which may cause severer blooms in winter and spring. However, a more stable water column due to atmospheric stilling and water level elevation mainly led to the increasing Chl*a*/TN and Chl*a*/TP in remaining months from 2005 to 2017, allowing algae to grow better. The results also indicated that water stability promotes algal growth mainly due to improved light availability. As atmospheric stilling is an aspect of global climate changes, this study would affect future algal bloom mitigation efforts in shallow lakes worldwide.

**Abstract:**

Algal blooms are environmental challenges confronting lakes worldwide and are significantly influenced by chlorophyll *a* yields per unit phosphorus (Chl*a*/TP), or nitrogen (Chl*a*/TN). Here, the influence of inter-annual hydrometeorological variations on Chl*a*/TP and Chl*a*/TN were evaluated in eutrophic shallow Lake Taihu, China. Our results demonstrated significant increases (*p* < 0.001) in both Chl*a*/TN and Chl*a*/TP from 2005 to 2017, and increased Chl*a* yields during the winter months were mainly correlated with higher water temperature and longer sunshine hours, which may cause severer blooms in winter and spring. In remaining months from 2005 to 2017, typical associations between atmospheric stilling (or water level elevation) and higher Chl*a* yields were observed. The results also indicate that atmospheric stilling and water level elevation significantly (*p* < 0.001) decreased background turbidity and promoted buoyant cyanobacterial biomass, alleviating phytoplankton light limitation. Given the subtropical location, eutrophic status, and high background turbidity of Lake Taihu, light may be the critical limiting factor for summer phytoplankton growth; thus, improved light availability would promote Chl*a* yields until self-shading caused further light limitations. If the mechanism is general, promoting the effect of atmospheric stilling on annual peak Chl*a* in shallow lakes may be greatly underestimated, and our finding will affect future bloom mitigation efforts in such systems.

## 1. Introduction

Nutrient enrichment and the associated algal blooms are widespread water quality issues in freshwater ecosystems [1]. Evidence indicates that algal blooms are increasing in frequency and intensity in lakes and reservoirs worldwide [1,2]. Algal blooms pose a range of ecological and socioeconomic problems, including threats to drinking water safety, access to water-based recreational activities, and detrimental effects on aquatic organisms [3,4]. In addition, toxins associated with algal blooms pose a range of risks to human health, from mild skin irritation to severe stomach upset and even death [5]. A range of actions have been proposed to combat algal blooms in freshwater ecosystems, including increasing the flushing rate, reducing external nutrient loading, and restoring macrophytes [6,7]. Current knowledge suggests that reducing nutrient inputs in the watershed is the most feasible way to control algal blooms owing to its predictable outcomes, long-term effectiveness, and relatively low cost [1,8,9]. In the majority of cases, reducing external nutrient loading generally resulted in a decline in phytoplankton biomass [6], which supports the implicit assumption that lake ecosystems impacted by eutrophication can be reverted to their original conditions by reducing nutrient inputs.

However, different results were observed in many large shallow lakes in the Middle–Lower Yangtze plains. A regional study showed that total nitrogen (TN) and total phosphorus (TP) concentrations increased by 8% and 3%, respectively, during the summer of 2018 (compared to summer 2008) in 27 large shallow lakes in this plain, while increases in Chl*a* concentration reached 240% [10]. In addition, monthly observations in Lake Taihu, a typical eutrophic large shallow lake in eastern China, revealed that TN has decreased continuously, while TP fluctuated from 2005 to 2017 [11], which may be due to the substantial restoration efforts since the drinking water crisis in May 2007 [4,12,13]. However, it is frustrating that chlorophyll *a* (Chl*a*) concentrations, a proxy for phytoplankton biomass, have multiplied in the northern bays of Lake Taihu during the last decade [11]. These findings indicate that Chl*a* yields per unit nutrient (as represented by Chl*a*/TN or Chl*a*/TP) in Lake Taihu and many other large shallow lakes in the Middle–Lower Yangtze plains have increased significantly in recent decades.

Chl*a* yields per unit nutrient, which reflect the nutrient use efficiency of the phytoplankton [14,15], play a critical role in determining algal biomass [16]. Understanding the factors influencing Chl*a* yields is conducive to identifying the conditions that are more susceptible to producing algal blooms under nutrient enrichment, and provides important information for formulating cost-effective eutrophication management strategies. For example, Huo et al. [17] have assessed the impact of meteorological (e.g., temperature) and geographic factors (e.g., altitude) on Chl*a* yields among Chinese lakes, which may provide scientific foundations for future categories-based nutrient management policies for lakes in China. Similarly, inter-annual changes in meteorological background, such as climate warming and atmospheric stilling, may also exert profound effects on Chl*a* yields. For example, warming can improve the photosynthesis-related enzyme activities, thus increasing Chl*a* yields [18]. Moreover, light is often the limiting factor for phytoplankton in nutrient-rich shallow lakes [16]. In such condition, the reduced background turbidity caused by atmospheric stilling may provide additional “light niches”, and thus promote the utilization efficiency of nutrients by algae [19]. However, inter-annual variations in Chl*a* yields and their driving factors have rarely been discussed or evaluated. Some studies have demonstrated typical statistical associations between climatic changes (e.g., climatic warming, atmospheric stilling, and more frequent occurrences of extreme precipitation) and increased algal biomass [20,21,22,23]. However, these studies have not clarified whether the promotion of algal biomass by climatic changes is due to increases in nutrient concentrations or to the enhancement of Chl*a* yields, and little is known about the relative importance of climate-related factors in driving inter-annual Chl*a* variations. In addition, previous study indicated that climatically modulated declines in wind speed may strongly promote algal growth in shallow lakes by facilitating the release of nutrients from the sediment [24]. However, wind speed is also closely related to the “light niches” of phytoplankton in shallow lakes, which have a profound effect on algal blooms. However, the effect of atmospheric stilling on light availability and algal growth is unclear, thus requiring further study.

The hydrometeorological conditions (e.g., water temperature, wind speed, water level and precipitation) at Lake Taihu have experienced pronounced changes over the past decades [20,25], which may exert profound effects on algal responses to nutrients. Thus, datasets from 2005 to 2017 in the northern bays of Lake Taihu were collected to (1) examine monthly dynamics of hydrometeorological factors, nutrients, Chl*a*, Chl*a*/TN, and Chl*a*/TP; (2) quantify the effects of inter-annual variations in the hydrometeorological factors on Chl*a* yields and identify the key influencing factors; and (3) provide information for future eutrophication management in shallow lakes in the context of climate change. This study may provide valuable information for bloom control in subtropical shallow lakes.

## 2. Materials and Methods

### 2.1. Study Area

Lake Taihu (30°55′40″–31°32′58″ N, 119°52′32″–120°36′10″ E) is located in the lower reaches of the Yangtze River, China. It is the third largest freshwater lake in China, with an area of 2338 km^2^, a catchment area of approximately 36,500 km^2^, a mean depth of 1.9 m, and a water residence time of approximately 220–309 days [3,4,26]. The Taihu catchment is strongly influenced by the East Asian monsoon and is located in a subtropical climate with four distinct seasons: spring (March–May), summer (June–August), autumn (September–November), and winter (December–February). The average air temperature and precipitation are approximately 16 °C and 1200 mm, respectively [27]. Intense wind-induced sediment re-suspension has been observed in Lake Taihu, and prevailing easterly–southeasterly winds dominate the wind direction. The ecological services in Lake Taihu include aquaculture, agricultural irrigation, flood control, tourism, and drinking water supply. The Taihu catchment is located in one of the most developed regions in China. The lake has undergone rapid eutrophication during the past several decades [3,4], and the massive nutrient loads from the basin combined with hot summer months (~30 °C) have resulted in frequent massive algal blooms [8]. 

### 2.2. Sampling and Monitoring 

**Figure 1:**
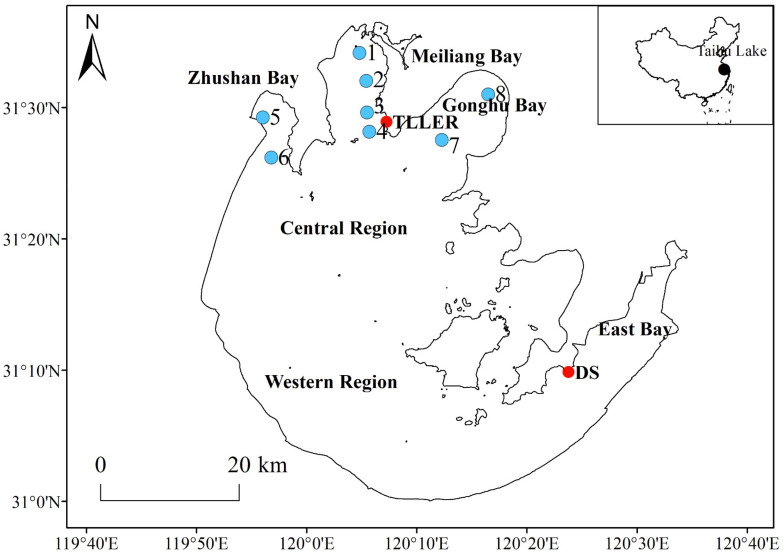
Map showing the spatial distribution of sampling sites, the Taihu Laboratory for Lake Ecosystem Research (TILLER), and Dongshan (DS) meteorological station.

In this study, eight sites with relatively homogeneous hydromorphological conditions in the northern bays of Lake Taihu, including Meiliang Bay, Zhushan Bay, and Gonghu Bay, were monitored monthly (Figure 1). From January 2005 to December 2017, water depth (WD) and Secchi depth (SD) were measured monthly using a SM-5 Portable Depth Sounder (Speedtech, River Falls, WI, USA) and Secchi disk at each sampling site. Water samples were also collected monthly at each site by mixing the surface (0.5 m below the surface), middle, and bottom (0.5 m above the bottom) layers of the water column. TN, TP, total dissolved nitrogen (TDN), total dissolved phosphorus (TDP), and Chl*a* were determined according to standard methods [28].

Daily meteorological datasets from Dongshan Station (DS, 31°24′ N, 121°27′ E) from 2005 to 2017 were obtained to determine the long-term trends in sunshine hours (SHH), average wind speed (AWS), and rainfall (RF) at Lake Taihu. These data were obtained from the China Meteorological Data Sharing Service System (http://data.cma.cn/). Daily observations of water level (WL, Wusong Datum system) and water temperature (WT) were also obtained from the Taihu Laboratory for Lake Ecosystem Research (TILLER, 31°42′ N, 120°22′ E).

### 2.3. Data Analyses

#### 2.3.1. Quantifying the Non-Algal Light Attenuation

**Figure 2:**
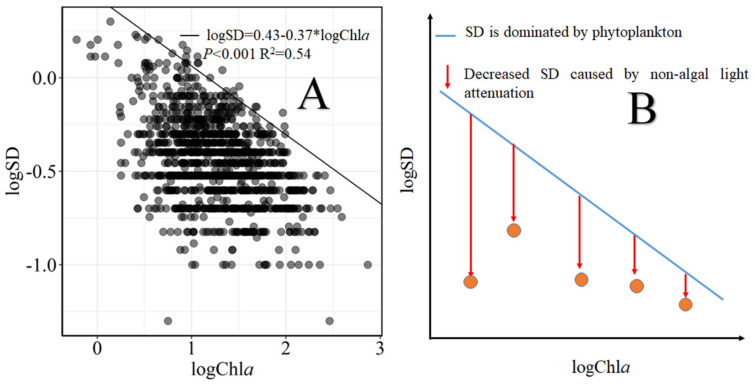
Graphs showing how to quantify non-algal light attenuation using the relationships between chlorophyll a (Chl*a*, μg/L) and Secchi depth (SD, m). Non-algal light attenuation was estimated by the difference between the predicted SD from the 95% quantile regression model (i.e., logSD = 0.43–0.37) * logChl*a* in (**A**) and the observed SD at the given derived Chl*a*. The datasets used for the scatter plot in (**B**) were for all the observations in the northern bays of Lake Taihu from 2005 to 2017.

In our study, we quantified non-algal light attenuation (NALA) in the northern bays of Lake Taihu from 2005 to 2017 using the method modified from Jones and Hubbart [29]. Specifically, the quantitative logSD-logChl*a* relationship, where water transparency is dominated by phytoplankton, was derived by 95% quantile regression model using all available datasets (Figure 2A). If observed SD was below the predicted SD derived from 95% quantile regression line at a given Chl*a*, this indicated that NALA was elevated, and the non-algal light attenuation was estimated by subtracting the observed SD from the predicted SD (Figure 2B). We note that the sites where the bottom was visible throughout the lake were excluded from the above-described analysis because actual SD was not measurable.

The quantile regression model can be viewed as an extension of classical least squares estimation of conditional mean models to estimate an ensemble of models for several conditional quantile functions [30]. Thus, the 95% quantile regression model can be used to estimate the rates of change for functions close to the upper boundary of a conditional distribution of responses, and it was implemented with *quantreg* package in the R software.

#### 2.3.2. Trend Analysis

Prior to conducting the trend analyses, the mean values of the monitored water quality variables (e.g., TN, TP, and Chl*a*) were calculated by averaging the data from the eight sampling sites for each month. The monthly mean values for the hydrometeorological variables (e.g., WL and AWS) were also calculated. The Mann-Kendall test (non-parametric) from the R package “*trend*” was then used with the averaged datasets to determine if significant temporal trends occurred in the variables. 

#### 2.3.3. Spearman Correlation Analysis

The spatial mean values for the monitored water quality variables at the eight sampling sites for each month and the monthly mean values for the hydrometeorological variables were also calculated. A Spearman correlation analysis was then conducted using SPSS 19.0 to identify the factors (i.e., WT, SHH, AWS, RF, and WL) that had significant effects on Chl*a*/TN (or Chl*a*/TP). The inner-annual variations in the hydrometeorological conditions (e.g., temperature) could significantly affect Chl*a*/TN (or Chl*a*/TP); thus, a Spearman correlation analysis was performed for each month from 2005 to 2017 to avoid the influence of inner-annual hydrometeorological variations on Chl*a* yields.

#### 2.3.4. Random Forest Model

The random forest model is a useful machine learning method that can determine non-linear relationships between response and explanatory variables by selecting explanatory variables according to their ability to minimize the sum of the error. To quantify the relative importance of each hydrometeorological factor on Chl*a* yields, the random forest model was utilized (using the function “*TreeBagger*” in Matlab) to determine the hierarchical relationship between the explanatory variables and Chl*a*/TN (or Chl*a*/TP). The purpose of this study was to analyze the influence of inter-annual hydrometeorological variations on Chl*a* yields; thus, the random forest model was conducted for each month from 2005 to 2017. 

## 3. Results

### 3.1. Trends in Environmental Factors and Chla Yields

**Figure 3:**
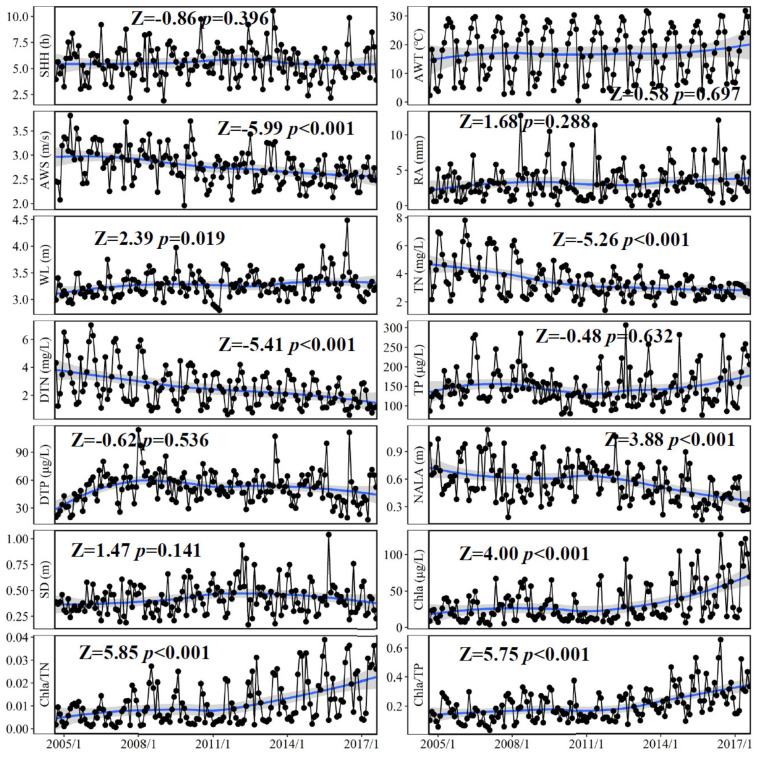
Trends in sunshine hours (SSH), average water temperature (WT), average wind speed (AWS), rainfall (RA), water level (WL), total nitrogen (TN), total dissolved nitrogen (TDN), total phosphorus (TP), total dissolved phosphorus (TDP), Secchi depth (SD), Chlorophyll *a* (Chl*a*), Chl*a*/TN, and Chl*a*/TP in the northern bays of Lake Taihu from January 2005 to December 2017. Z is a statistical parameter of Mann-Kendall test analysis, Z > 0 (or Z < 0) and *p* < 0.05 indicate a significant increasing (or decreasing) trend. Blue shaded area represents the fitting curve estimated from locally weighted regression model.

Significant decreasing trends (*p* < 0.001) in AWS, TN, TDN, and NALA were observed in the northern bays of Lake Taihu from 2005 to 2017, while significant (*p* < 0.001) increases in Chl*a*, Chl*a*/TN, and Chl*a*/TP were observed from 2005 to 2017 (Figure 3). For the remaining variables (i.e., SHH, AWT, RA, WL, TP, TDP, and SD), no significant trends (*p* > 0.05) were observed during the study period. 

### 3.2. Chla Yields Driving Force

**Figure 4:**
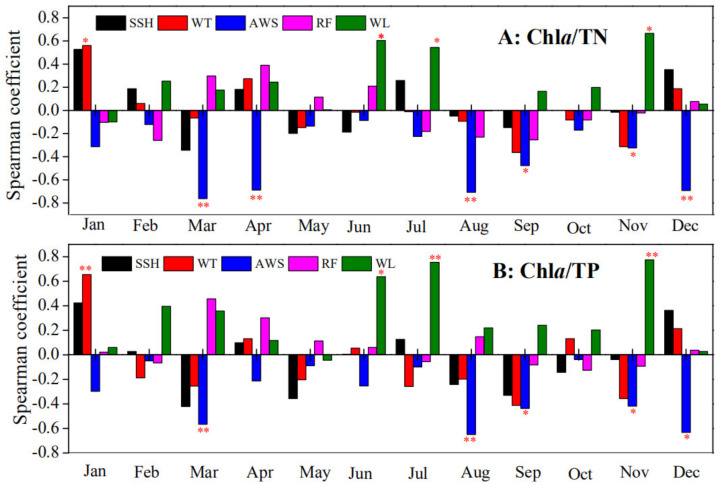
Results of the Spearman correlation analysis between hydrometeorological variables (sunshine hours, SHH; water temperature, WT; average wind speed, AWS; rainfall, RF; and water level, WL) and chlorophyll *a* yields (**A**): Chl*a*/TN. (**B**): Chl*a*/TP) for each month from 2005 to 2017 in the northern bays of Taihu Lake. Red ** and * indicate significance levels of *p* < 0.01 and *p* < 0.05, respectively.

Spearman correlation analysis showed that AWS was significantly (*p* < 0.05) negatively correlated with Chl*a*/TN in March, April, August, September, November, and December. Moreover, significant (*p* < 0.05) negative correlations were also observed between AWS and Chl*a*/TP in March, August, September, November, and December. The Chl*a* yields (both Chl*a*/TN and Chl*a*/TP) were significantly (*p* < 0.05) positively correlated with WL in June, July, and November from 2005 to 2017. Significant (*p* < 0.05) positive correlations between AT and Chl*a*/TN (or Chl*a*/TP) were also observed in January (Figure 4). 

**Figure 5:**
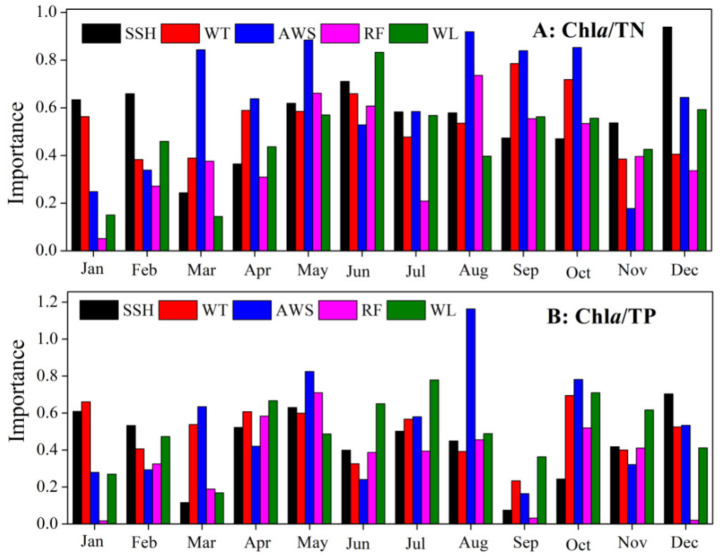
The importance of each explanatory variable (sunshine hours, SHH; water temperature, WT; average wind speed, AWS; rainfall, RF; and water level, WL) on variations in Chla/TN (or Chla/TP) for each month from 2005 to 2017 in the northern bays of Taihu Lake, based on the random forest model.

Among the explanatory variables in the random forest model, SHH had the largest influence on inter-annual variations in Chl*a*/TN during November, December, January, and February. During the remaining months, AWS (or WL) had the largest influence on Chl*a*/TN variations. The inter-annual variations in Chl*a*/TP were mostly affected by SHH in December and February. Moreover, WT had the largest influence on Chl*a*/TP in January. During the remaining months, AWS (or WL) had the largest influence on inter-annual Chl*a*/TP variations (Figure 5).

## 4. Discussion

### 4.1. Key Factors Influencing Chla Yields

The most common factors that influence Chl*a* yields are temperature and light conditions [17,31]. For example, in Lake Võrtsjärv and Lake Peipsi in Estonia, nutrient reduction was followed by increases in phytoplankton biomass [6,32]. In these studies, the authors suggested that climatic warming was likely to have promoted the algal responses to nutrients. In our study, the promoting effect of improved photothermal conditions on Chl*a* yields also occurred during the winter months (Figure 4 and Figure 5). It is reasonable that increased AWT and longer SHH increased Chl*a* yields in Lake Taihu during winter months because algal growth in these months was generally limited by poor light availability and low temperature [21]. However, in the remaining months from 2005 to 2017, the limited inter-annual variation in AWT decreased the detectability of its influence on Chl*a* yields (Figure 3). The typical associations were observed between decreased AWS (or increased WL) and the enhanced Chl*a* yields in the remaining months (March to November) from 2005 to 2017 (Figure 4 and Figure 5). Obviously, wind has a strong influence on horizontal transport of phytoplankton, especially for Lake Taihu, where floating cyanobacteria dominated the algal community [33]. However, negative correlations between Chl*a* yields and wind speed in our study may not be ascribed to the wind-induced horizontal transport of algae. In Lake Taihu, the dominant wind direction is east–southeast [3]; in this condition, lower Chl*a* yields of nutrients in depth-integrated samples were expected in the northern bays with the atmospheric stilling, as fewer algae should be blown to the northern lake. This result indicates the wind speed decline increased Chl*a* yields mainly by promoting algal growth. 

**Figure 6:**
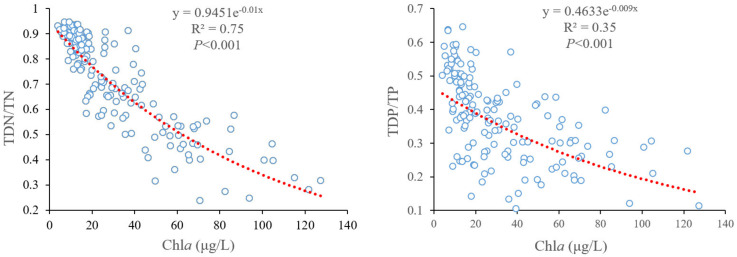
Regression analyses between chlorophyll *a* (Chl*a*, μg/L) and the ratio of total dissolved nitrogen to total nitrogen (TDN/TN), and the ratio of total dissolved phosphorus to total phosphorus (TDP/TP) in the northern bays of Lake Taihu from 2005 to 2017.

The results obtained in this study indicate that typical associations between decreasing wind speed (or elevated water level) and higher Chl*a* yields may be related to phytoplankton light limitations. On the one hand, light availability may be a critical limiting factor for algal growth in Lake Taihu. Specifically, higher Chl*a* values were observed with decreasing TDN/TN (or TDP/TP) because algal growth consistently converts dissolved nutrients into particulate nutrients stored in the phytoplankton (Figure 6). Based on the regression equations between Chl*a* and TDN/TN (or TDP/TP) (Figure 6), when Chl*a* reached 100 µg/L, the values of TDN/TN and TDP/TP were 35% and 19%, respectively. These results imply that dissolved nutrients could support further increases in algal biomass, and that non-nutrient factors limited the maximum algal growth potential in Lake Taihu. In turbid and nutrient-rich shallow lakes, light is often the most important factor that limits algal biomass [34,35,36,37,38]. The mechanism is that high background turbidity (i.e., fractional light absorption by abiotic substances, including water) caused by intense sediment re-suspension and increasing self-shading effect often limits algal growth before the standing crop reaches the levels permitted by the nutrient concentrations [16]. Lake Taihu is characterized by intense wind-induced sediment re-suspension, with mean total suspended solids of 56.8 mg/L during the winter months [39], indicating that the background turbidity of Lake Taihu is very high. Therefore, given its subtropical location, eutrophic nutrient status, and high background turbidity (Figure 3), we suggest that light availability is a critical limiting factor for phytoplankton biomass in Lake Taihu.

**Figure 7:**
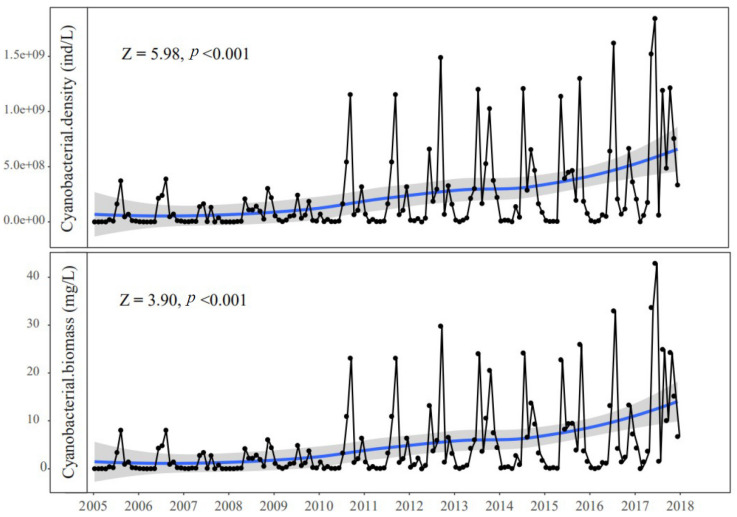
Trend in cyanobacterial density and biomass in the Meiliang Bay of Lake Taihu from January 2005 to December 2017. The blue shaded area indicates the standard error of the estimate, Z is a statistical parameter of Mann-Kendall test analysis, Z > 0 and *p* < 0.05 indicate a significant increasing trend. Blue shaded area represents the fitting curve estimated from locally weighted regression model. The datasets used in this figure were acquired from Guo et al. [26].

On the other hand, a nearly 45% decrease in the difference between the predicted SD from the 95% quantile regression model and the observed SD at given Chl*a* was observed in the northern bays of Lake Taihu from 2005 to 2017, indicating that background turbidity had decreased considerably during our study period mainly due to atmospheric stilling and elevated water levels. Meanwhile, as shown in Guo et al. [26], increasing trends (*p* < 0.001) in both cyanobacterial cells and biomass were observed from 2005 to 2017 in Meiliang Bay on Lake Taihu (Figure 7). This may be due to the fact that a calmer water column caused by decreasing wind speeds or increasing water level is beneficial to the growth of buoyant cyanobacteria (e.g., *Microcystis* spp.) [40,41]. A larger cyanobacterial standing crop indicates that light limitations on algal growth will be alleviated because more phytoplankton cells will float to the surface, where they have better access to light [21,42,43]. If the system is light-limited, a substantial decrease in background turbidity and increased buoyant cyanobacterial cells could provide an additional “light niche” for enhancing Chl*a* yields until the algal biomass decreased the available underwater light, causing self-shading and further light limitations [19,38,44]. In addition, a calmer water column generally results in higher ratios of dissolved nutrients in Lake Taihu, which may also promote Chl*a* yields. It seems reasonable to assume that atmospheric stilling and elevation of the water level increased Chl*a* yields by improving algal light availability. However, the caveat is causality cannot be inferred from statistical analyses; thus, further research via enclosure experiments is required to verify the cause–effect connections among decreases in wind speed (or increases in water level), phytoplankton light availability, and Chl*a* yields.

### 4.2. Implications for Management

Traditional robust positive linear relationships between log-transformed algal biomass and nutrient concentrations (nitrogen and phosphorus) form a logical basis for prioritizing nutrient input constraints as a “bottom line” bloom mitigation strategy [8,45]. Thus, reducing nutrients in the water column is generally expected to decrease Chl*a* concentrations [6]. However, Chl*a* yields increased significantly in the northern bays of Lake Taihu from 2005 to 2017, and increasing algal biomass has been observed with fluctuating TP and decreasing TN concentrations. Our results indicated that inter-annual changes in hydrometeorological conditions have a significant promoting effect on Chl*a* yields. On the one hand, warming and longer sunshine hours in winter months from 2005 to 2107 increased Chl*a* yields, which might support higher concentrations of overwintering phytoplankton cells [15], resulting in early initiation, higher biomass, and longer duration of algal blooms during the spring months [46]. However, considering the relatively fast phytoplankton reproduction rate, improved photothermal conditions during the winter months may not be the main driver of inter-annual Chl*a* variations during hot summer months in Lake Taihu.

The statistical analyses indicate that the increasing Chl*a* yields during the spring, summer, and autumn months from 2005 to 2017 in northern Lake Taihu were mainly related to decreases in wind speed and increases in water level (Figure 4 and Figure 5). The mechanisms may involve (1) high background turbidity and surplus nutrients that made light the critical limiting factor of Chl*a*, (2) decreasing wind speeds and increasing water levels that alleviated the light limitations by decreasing background turbidity and increasing floating cyanobacterial biomass [19,38]. As a result, nutrients were utilized by the algae more efficiently until increasing algal turbidity induced further light limitation. In reality, Scheffer [16] has suggested that the total turbidity is independent of the background turbidity because algae grow to reach a fixed maximum self-shading when phytoplankton are light-limited. We believe this might also be the key mechanism due to which the SD in the northern bays of Lake Taihu did not significantly change under decreasing background turbidity with wind speed decline and water level elevation from 2005 to 2017. These findings may have important implications for eutrophication management in Lake Taihu. Firstly, caution should be used in practices that might reduce levels of background turbidity (e.g., water level elevation), unless external nutrient loadings are well controlled [19,47], because background turbidity may be preventing algal blooms if phytoplankton is light-limited. Secondly, algal biomass may exhibit limited response to nutrient reduction until nutrients become the limiting factor of algae in Lake Taihu; thus, it may take a long time to achieve good efficacy with nutrient loading reductions in controlling the blooms. Thirdly, inter-annual changes in the hydrometeorological factors seem to run significantly counter to the reductions in nutrient loading rather than reinforcing re-oligotrophication; thus, stricter nutrient thresholds and reduction strategies will be required in Lake Taihu under calmer climatic conditions in the future. In mid-low latitudes, surface wind speed has been undergoing continuous decline since 1960 (i.e., global atmospheric stilling) [48]. Thus, calmer water columns due to atmospheric stilling may also lead to increased Chl*a* yields in other subtropical eutrophic shallow lakes [34,35,36,37,38]. However, the modulating effects of climatic warming on nutrient limitation of algal growth in shallow turbid lakes has always been the focus in previous studies. Therefore, we believe that our study makes a significant contribution to the literature because we found that wind speed decline, not temperature, is like to be the key factor influencing annual peak Chl*a* in eutrophic shallow lakes, and this will affect future algal bloom mitigation efforts in such systems.

## 5. Conclusions

Current study revealed significant increases (*p*< 0.001) in both Chl*a*/TN and Chl*a*/TP from 2005 to 2017, indicating an increasing nutrient use efficiency of the phytoplankton during the study period. Further analysis indicated warming and longer sunshine hours boost Chl*a* yields in winter months, which may result in higher concentrations of overwintering phytoplankton cells and severer of algal blooms in early spring months. In addition, current study also demonstrated that increasing Chl*a* yields were typically associated with atmospheric stilling (or water level elevation) in the months with relatively higher temperature. The specific mechanisms including: (1) the subtropical location and eutrophic nutrient status of Lake Taihu makes light availability is a critical limiting factors for algal growth; (2) atmospheric stilling and water level elevation have significantly improved algal “light niches” by decreasing non-algal turbidity and prompting buoyant cyanobacteria distributed in the surface water layer. As a result, increased Chl*a* yields were observed until the self-shading returns light limitation. This might also be the key mechanism due to which the SD in Lake Taihu during our study period did not change significantly under decreasing wind speed and TN levels, and increasing water level. If the mechanism is general, these results demonstrate a new global factor (i.e., wind speed decline) is likely to run counter significantly to nutrient reductions in large shallow lakes. Thus, stricter nutrient thresholds are needed in large shallow lakes to offset the positive contribution of wind speed decline to algal blooms. 

## Data Availability

Not applicable.
